# Molecular detection of persistent postoperative circulating tumour cells in stages II and III colon cancer patients via multiple blood sampling: prognostic significance of detection for early relapse

**DOI:** 10.1038/bjc.2011.40

**Published:** 2011-02-22

**Authors:** C-Y Lu, Y-H Uen, H-L Tsai, S-C Chuang, M-F Hou, D-C Wu, S-H Hank Juo, S-R Lin, J-Y Wang

**Affiliations:** 1 Division of Gastroenterology, Department of Internal Medicine, Kaohsiung Medical University Hospital, Kaohsiung Medical University, Kaohsiung, Taiwan; 2 Department of Internal Medicine, Faculty of Medicine, College of Medicine, Kaohsiung Medical University, Kaohsiung, Taiwan; 3 Division of General Surgery, Department of Surgery, Chi-Mei Medical Center, Tainan, Taiwan; 4 Department of Medical Research, Chi-Mei Medical Center, Tainan, Taiwan; 5 Department of Electrical Engineering, Southern Taiwan University, Tainan, Taiwan; 6 Department of Emergency Medicine, Kaohsiung Municipal Hsiao-Kang Hospital, Kaohsiung Medical University, Kaohsiung, Taiwan; 7 Program of Bachelor of Health Beauty, School of Medical and Health Sciences, Fooyin University, Kaohsiung County, Taiwan; 8 Division of Hepatobiliary Surgery, Department of Surgery, Kaohsiung Medical University Hospital, Kaohsiung Medical University, Kaohsiung, Taiwan; 9 Department of Surgery, Faculty of Medicine, College of Medicine, Kaohsiung Medical University, Kaohsiung, Taiwan; 10 Cancer Center, Kaohsiung Medical University Hospital, Kaohsiung Medical University, Kaohsiung, Taiwan; 11 Department of Medical Genetics, Faculty of Medicine, College of Medicine, Kaohsiung Medical University, Kaohsiung, Taiwan; 12 Department of Medical Research, Fooyin University Hospital, Pingtung County, Taiwan; 13 Department of Medical Technology, School of Medical and Health Sciences, Fooyin University, Kaohsiung County, Taiwan; 14 Division of Gastrointestinal and General Surgery, Department of Surgery, Kaohsiung, Taiwan; 15 Graduate Institute of Medicine, Kaohsiung Medical University, Kaohsiung, Taiwan

**Keywords:** molecular detection, colon cancer, postoperative early relapse, circulating tumour cells, UICC stage II/III

## Abstract

**Background:**

The purpose of this study was to detect postoperative persistent circulating tumour cells (CTCs) in stages II and III colon cancer patients undergoing curative resection and so identify a subgroup of patients who are at high risk for early relapse.

**Methods:**

Four mRNA molecular markers including human telomerase reverse transcriptase, cytokeratin-19, cytokeratin-20, and carcinoembryonic antigen (CEA) mRNA were used to detect CTCs in 141 stages II and III colon cancer patients undergoing curative resection to determine the significance of CTCs in postoperative early relapse.

**Results:**

Out of 141 patients, postoperative early relapse and non-early relapse/no relapse was found in 48 (34.0%) patients and 93 (66.0%) patients, respectively. Univariately, postoperative early relapse was significantly correlated with lymph node metastasis (*P*=0.025), vascular invasion (*P*=0.002), perineural invasion (*P*=0.001), laparoscopic surgery (*P*=0.019), high postoperative serum CEA levels (*P*=0.001), and presence of persistent postoperative CTCs (*P*<0.001). Using a multivariate proportional hazards regression analysis, the presence of perineural invasion (*P*=0.034; HR, 1.974; 95% CI: 1.290–3.861), high postoperative serum CEA levels (*P*=0.020; HR, 2.377; 95% CI: 1.273–4.255), and the presence of persistent postoperative CTCs (*P*<0.001; HR, 11.035; 95% CI: 4.396–32.190), were demonstrated to be independent predictors for postoperative early relapse. Furthermore, the presence of persistent postoperative CTCs was strongly correlated with a poorer disease-free and overall survival (both *P*<0.001).

**Conclusions:**

This study suggests that molecular detection of persistent postoperative CTCs is a prognostic predictor of early relapse in UICC stage II/III colon cancer patients, and thus could help to define patients with this tumour entity for an enhanced follow-up and therapeutic program.

Colorectal cancer (CRC) is a common neoplasia in the world and also a major cause of cancer-related death (Frederiksen *et al*, 2010; Jemal *et al*, 2010). Although there have been significant improvements in the treatment of advanced CRC patients, such as the use of adjuvant chemotherapy, individuals with advanced disease still have poor prognosis (Gallagher and Kemeny, 2010). The use of adjuvant chemotherapy after curative surgery has been demonstrated to improve outcome among patients with high-risk stage II and stage III CRC (Andre *et al*, 2004; Wilkinson *et al*, 2010). Surgery remains the mainstay of therapy, but recurrence after curative surgery of CRC occurs at a constant rate according to the stage of the disease, and the more advanced the stage, the greater the recurrent rate (Korner *et al*, 2005). The recurrence rates in patients with stages I, II, and III CRC are 3.7, 13.3, and 30.8%, respectively (Kobayashi *et al*, 2007). The recurrence of CRC is also for the most part a time-related phenomenon, as 40–50% of recurrences become apparent within the first year after initial resection, and 90% within the first 4 years (Longo and Johnson, 2002). Recurrences in stages II and III patients showed a rapid increase for the first 3 years, whereas the rate of those with stage I cancer increased gradually and constantly for 5 years (Kobayashi *et al*, 2007). Early relapse in CRC patients is attributed mainly to the higher malignant entity (such as an unfavourable genotype, deeper tumour invasion, lymph node metastasis, and advanced cancer stage) and poor response to chemotherapy. There was a consistent trend of poorer survival rates in cases of earlier relapse. Therefore, it is valuable that we could detect the significant predictive factors of postoperative early relapse.

For many decades, the depth of tumour invasion, local lymph node involvement, and the presence or absence of distant metastasis have been used as major prognostic factors to predict the postoperative relapse in American Joint Commission on Cancer/International Union Against Cancer (AJCC/UICC) colon cancer patients (Edge *et al*, 2010). Efforts have concentrated on the early detection of recurrent tumours to ensure adequate and effective treatment to improve patient's prognosis (Rodriguez-Moranta *et al*, 2006). The identification of specific colon tumour-associated molecular markers and the development of robustly accurate assay method for effective disease monitoring would significantly advance the early diagnosis of recurrence and subsequently the effective treatment (Nannini *et al*, 2009). Undetected micrometastases contribute to the failure of primary curative surgery of CRC (Zhang *et al*, 2005; Steinert *et al*, 2008). Therefore, the detection of tumour shed cells in bloodstream is very important in early recognition of postoperative CRC patients necessitating further optimal therapy (Rahbari *et al*, 2010).

The concept of circulating tumour cells (CTCs) in the peripheral blood of cancer patients was documented by Ashworth in 1869. With the advance of molecular biology and refined techniques, the identification of CTCs via nucleic acid-based methodology and polymerase chain reaction (PCR) has been proven to be a useful tool in the detection of occult metastases (Allan and Keeney, 2010). The conventional PCR, reverse-transcriptase PCR, or real-time quantitative PCR (Q-PCR) assays permit sensitive detection of CTCs, but only one molecular marker can be checked at a time. As a result of the heterogeneity of genetic biomarker expression in blood, a multi-marker assay is regarded as more sensitive, time-saving, and cost-effective than a single-marker assay (Yeh *et al*, 2006; Allan and Keeney, 2010). Our previously developed membrane-array method using multi-marker assay can detect CTCs as few as five cancer cells in 1 ml of peripheral blood (approximately one tumour cell per 10^6^ white blood cells) in CRC patients (Wang *et al*, 2006; Yeh *et al*, 2006), and it is applied to the current investigation for the postoperative surveillance of colon cancer patients.

So far, it remains a challenge to use proper molecular markers for the detection, and then give optimal treatment of postoperative early relapse in UICC stages II and III CRC patients (Allan and Keeney, 2010; Rahbari *et al*, 2010). The aim of this study was to determine the prognostic significance of persistent CTCs via multiple blood sampling in the detection of postoperative early relapse in stages II and III colon cancer patients, and thus help to define patients with this tumour entity for an enhanced follow-up and therapeutic program.

## Patients and methods

### Patients and samples collection

Between January 2004 and August 2008, a total of 381 AJCC/UICC stages II and III colon cancer patients who underwent curative resection from the Kaohsiung Medical University Hospital were reviewed under the retrospective observational study. Of 381 patients, 50 patients with other malignancies, those lost to follow-up or followed up for <1 year, and those having had surgical mortality or positive surgical resection margin for tumour invasion were excluded. Curative surgery was defined as any gross residual tumour that did not remain in the surgical bed and in which the surgical resection margin was pathologically negative for tumour invasion. To decrease the false-negative rate of CTCs in predicting postoperative relapse, only cDNA from multiple peripheral blood samples that could be obtained was enrolled, therefore, 190 patients were excluded because of incomplete multiple blood sampling postoperatively. Finally, 141 sets of cDNA in 1 and 4 weeks after radical resection were entered into this study. There was no statistical difference in the overall survival between the 141 studied subjects and the 190 discarded ones (*P*=0.245). The development of new postoperative recurrent or metastatic lesions was defined as postoperative relapse. Early relapse was defined as local recurrence (tumour growth restricted to the anastomosis or the region of the primary operation) or distant metastasis (distant metastasis or diffuse peritoneal seeding) within 1 year after radical resection. Postoperative surveillance consisted of medical history, physical examination, and laboratory studies including serum carcinoembryonic antigen (CEA) levels every 3 months. Abdominal ultrasonography or computed tomography was performed every 6 months, and chest radiography and total colonoscopy were performed once a year. However, patients with significantly elevated serum CEA level lasting >4–6 months, abdominal or chest-computed tomography would be done before the annual check-up. Patients were followed up at 3-monthly intervals for 2 years and 6-monthly intervals thereafter. We intensively followed up these enrolled patients until December 2009, and the median follow-up time was 40 months (range 16–72 months). Of these patients, 72 cases developed postoperative relapse during the follow-up period, and 48 cases were classified into early relapse. Patients diagnosed as either high-risk stage II or III would receive adjuvant chemotherapy. Patients with risk factors including tumour poorly differentiated, T4 stage, tumour perforation/obstruction, number of lymph nodes examined <12, or lymphatic/vascular invasion were considered as high-risk stage II cases. Intravenous 5-fluorouracil (5-FU)/leucovorin (LV) or oral tegafur-uracil (UFUR)/LV was administrated to high-risk stage II colon cancer patients, while 5-FU/LV/oxaliplatin or oral capecitabine was administrated to stage III colon cancer patients. The duration of adjuvant chemotherapy was given for half a year.

Circulating tumour cells in peripheral blood of these 141 patients were detected using our previously constructed membrane-array method (Wang *et al*, 2006; Yeh *et al*, 2006). Briefly, a 4-ml sample of peripheral blood was obtained from each CRC patient postoperatively (1 and 4 weeks after operation) for total RNA isolation. To prevent contamination of epithelial cells, peripheral blood samples were obtained through a catheter inserted into a peripheral vessel, and the first 5 ml of blood were discarded. The definition of persistent CTCs in studied subjects was defined as detectable CTCs by membrane-array method postoperatively (both 1 and 4 weeks after operation). Written informed consent was obtained from each subject and/or guardian. Sample acquisition and subsequent use were also approved by the hospital's institutional review board. Preoperative staging methods included chest X-ray, abdominal ultrasound, bone scan, and abdominal-computed tomography. Clinical stage and pathological features of primary tumours were defined according to the criteria of the AJCC/UICC (Edge *et al*, 2010).

### Detection of serum CEA

An additional 3-ml peripheral blood samples from 141 colon cancer patients were obtained <1 week before operation (preoperative) and 4 weeks after operation (postoperative). Serum CEA levels were determined by means of an enzyme immunoassay test kit (DPC Diagnostic Product Co., Los Angeles, CA, USA) with the upper limit of 5 ng ml^–1^ defined as normal according to the manufacturers of the kits used.

### mRNA isolation and first strand cDNA synthesis

Total RNA was extracted from the fresh whole blood of preoperative/postoperative CRC patients using a QIAmp RNA Blood Mini Kit (QIAGEN Inc., Valencia, CA, USA) according to the manufacturer’s instructions. The RNA concentration was determined spectrophotometrically on the basis of absorbance at 260 nm. First strand cDNA was synthesised from total RNA by using a RT–PCR kit (Promega Corp., Madison, WI, USA).

### Membrane arrays

The procedure of the membrane-array method for the detection of CTC-related mRNA molecular markers was performed according to our previous study (Wang *et al*, 2006; Yeh *et al*, 2006). Patients overexpressing all four molecular markers by membrane-array methods in peripheral blood samples obtained postoperatively (both 1 and 4 weeks after operation) were considered as positive results of CTCs. In our previous investigation, the sensitivity limit of this technique was established at approximately one tumour cell per 10^6^ white blood cells (five cells per 1 ml blood) (Wang *et al*, 2006; Yeh *et al*, 2006).

### Statistical analysis

All data were statistically analysed using the Statistical Package for the Social Sciences, version 12.0 (SPSS Inc., Chicago, IL, USA). A *P-*value <0.05 was considered statistically significant. Receiver operating characteristics curve analyses were carried out to determine the sensitivity and specificity for each mRNA marker of membrane arrays (Wang *et al*, 2006; Yeh *et al*, 2006). The cut-off values for each mRNA marker were set at points representing the highest accuracy of analysis (minimal false-negative and false-positive results). The difference between data obtained by membrane array and real-time Q-PCR was calculated by using linear regression and Pearson's correlation. The univariate analysis of clinicopathologic features and presence of CTCs between the two groups (relapse group *vs* non-relapse group) was compared using the *χ*^2^ test. Independent prognostic factors for postoperative relapse were determined using a multivariate Cox proportional hazards regression analysis. The disease-free survival rates and overall survival rates were calculated by the Kaplan–Meier method, and the differences in survival rates were analysed by the log-rank test.

## Results

The average age of 141 patients was 64.1 years (range 30–88 years, Table 1). A total of 58 tumours (41.1%) were at the right-sided colon and 83 (58.9%) at the left-sided colon. With regard to the histological type of these tumours, 10 (7.1%) were well differentiated, 103 (73.0%) were moderately differentiated, and 28 (19.9%) were poorly differentiated carcinoma. In view of clinicopathologic characteristics of these 50 (35.5%) UICC stage II and 91 (64.5%) UICC stage III colon cancer patients, 74 of 141 patients (52.5%) were identified to have vascular invasion, and 78 of 141 patients (55.3%) were found to have perineural invasion. High serum CEA level (⩾5 ng ml^–1^) was observed in 85 (60.3%) of preoperative and 47 (33.3%) of postoperative colon cancer patients, and persistent postoperative CTCs were detected in 51 (36.2%) of 141 colon cancer patients.

Table 2 discloses the correlation between persistent postoperative CTCs and clinicopathologic features of 141 stages II and III colon cancer patients. Under univariate analysis, depth of invasion (*P*=0.029), vascular invasion (*P*<0.001), perineural invasion (*P*=0.002), high preoperative serum CEA level (*P*=0.025), and high postoperative serum CEA level (*P*<0.001) were significant in the presence of persistent postoperative CTCs. If multivariate logistic regression analysis was used, the presence of vascular invasion (*P*=0.019; HR: 2.732, 95% CI: 1.176–6.394) and high postoperative serum CEA level (*P*=0.013; HR: 2.712, 95% CI: 1.230–5.988) independently predicted the presence of persistent postoperative CTCs (Table 3).

**Figure 1:**
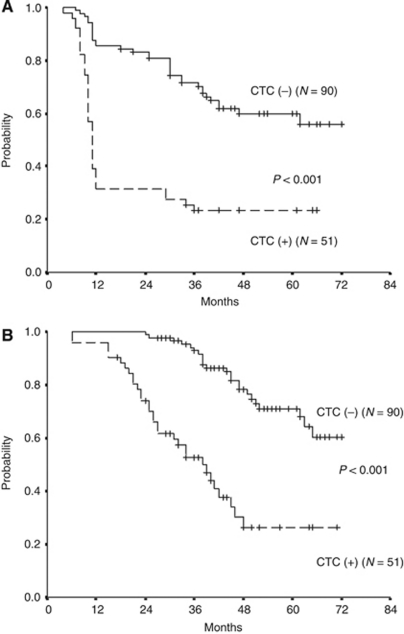
(**A**) Cumulative disease-free survival of 141 stages II and III colon cancer patients according to the persistent postoperative presence of CTCs. Colon cancer patients with persistent CTCs in peripheral blood showed a significantly poorer disease-free survival than those without CTCs in peripheral blood (*P*<0.001). (**B**) Cumulative overall survival of 141 stages II and III colon cancer patients according to the persistent postoperative presence of CTCs. Colon cancer patients with persistent postoperative CTCs in peripheral blood showed a significantly poorer overall survival than those without CTCs in peripheral blood (*P*<0.001).

Table 4 shows the correlation between clinicopathologic features and postoperative relapse of 141 stages II and III colon cancer patients. Using univariate analysis, we found that lymph node metastasis (*P*=0.025), vascular invasion (*P*=0.002), perineural invasion (*P*=0.001), type of surgery (*P*=0.019), high postoperative serum CEA level (*P*=0.001), and presence of persistent postoperative CTCs (*P*<0.001) were significantly correlated to postoperative early relapse. Using a multivariate Cox proportional hazards regression analysis, the presence of perineural invasion (*P*=0.034; HR: 1.974, 95% CI: 1.290–3.861), high postoperative serum CEA level (*P*=0.020; HR: 2.377, 95% CI: 1.273–4.255), and the presence of persistent CTCs (*P*<0.001; HR: 11.035, 95% CI: 4.396–32.190) were demonstrated to be independent predictors for postoperative early relapse (Table 5). The median disease-free time and overall survival time of the 141 studied patients were 45 and 62 months, respectively. Furthermore, both disease-free and overall survival rate in patients with persistent CTCs (Figure 1) were significantly lower than in those without persistent CTCs using a log-rank test (both *P*<0.001).

## Discussion

It is notable to find that as many as 40–50% of recurrent CRC develop within the first 1 year after ‘so-called’ curative surgery (Longo and Johnson, 2002), suggesting that incomplete pathological factors or undetected micrometastases actually exist and may have an important role in subsequent relapse (Zhang *et al*, 2005; Steinert *et al*, 2008). Tumour relapse after curative resection of CRC is attributed to tumour cell dissemination, currently underdetermined by standard TNM classification. There is often significant heterogeneity of tumour behaviour and variable patient outcome, which is unexplainable by pathologic factors alone. That is to say, not all CRC patients with early UICC stage tumours are cured and not all patients with advanced ones die from their disease. This resulted in a number of efforts to develop more accurate staging protocols to refine subsets appropriate for targeted therapy (Greene, 2006; Roukos *et al*, 2007; Sun *et al*, 2009).

Disseminating tumour cells were suggested to shed from the primary neoplasm into circulation before or during operation (Yamaguchi *et al*, 2000). Accumulated reports have documented the presence of CTCs in pre- or postoperative CRC patients, and this would probably lead to postoperative relapse among these patients (Yamaguchi *et al*, 2000; Molnar *et al*, 2001; Zhang *et al*, 2005; Wong *et al*, 2009; Peach *et al*, 2010). The fact that about half of CRC patients relapse within 1 year after curative surgery emphasises the urgent need for finding practical diagnostic tools to detect CTCs to improve prediction of recurrence with conventional UICC stage. This study has demonstrated that stages II–III colon cancer patients identified with persistent postoperative CTCs using multi-marker membrane-array method exhibit higher incidence of postoperative early relapse and poorer disease-free and overall survival rate. Consistent results are obtained from a recently published meta-analytic report by (Rahbari *et al*, 2010), whose analysis enrolled a total of 3094 CRC patients from 36 eligible studies. Pooled analysis shows that detection of CTCs in the peripheral blood indicates poorer recurrence-free survival (HR=3.24, 95% CI: 2.06–5.10) and overall survival (HR=2.28, 95% CI: 1.55–3.38). Our previous report (Uen *et al*, 2008) studied 438 stages I–III CRC patients undergoing curative resection, and 137 of them had presence of both pre- and postoperative CTCs that was strongly correlated with poorer relapse-free survival rates in any of colorectal, colon or rectal cancer groups. Early relapse cases had significantly lower overall survival rates than non-early relapse cases either in colon cancer or in rectal cancer patients (Tsai *et al*, 2009). Consequently, through multiple blood sampling, this study emphasised that persistent postoperative CTCs (lasting for 4 weeks postoperatively) might have a role in the early identification of postoperative early relapse in colon cancer patients, and more intensive therapies may be considered among these patients.

On the contrary, some reports have conflicting results regarding the prognostic role of CTCs in CRC patients (Bessa *et al*, 2001, 2003). The major cause of discrepancy between those reports and ours may be that only a single molecular marker was used to check CTCs in pre- or postoperative CRC patients in their studies, which was thought to be more underestimated compared with a multi-marker method (Yeh *et al*, 2006; Allan and Keeney, 2010). In addition, small and inhomogeneous patient groups were also attributed to the aetiology of discrepancy between those reports and our results. In fact, there are 22.9% (11 out of 48) false-negative and 15.1% (14 out of 93) false-positive rates of identifying early relapses in postoperative stages II–III colon cancer patients using our multi-marker membrane-array method, suggesting that there is room for the refinement of this method. The false-negative of our multi-marker array method in predicting postoperative early relapse, at least in part, might result from CTCs intermittently spreading into the bowel bloodstream, and the heterogeneous biologic nature of the tumour itself which cannot overexpress all four molecular markers (Wang *et al*, 2006). In our study, the definition of early relapse is clinically detectable tumour within 1 year after operation. Fourteen patients (14 out of 93, 15.1%) had false-positive results, which meant they had positive postoperative CTCs but did not have early relapse during follow-up. One of the explanations is inadequate follow-up time to detect recurrent lesions. However, some of them (24 out of 93, 25.8%) did have tumour relapse >1 year after the operation. Furthermore, few cancer cells shed into the bloodstream can establish a successful metastasis, and that also can make a false-positive result (Wang *et al*, 2006). Therefore, extending the follow-up period and/or creating new probes to replace the present one may improve the false-positive/negative rates of our membrane-array method.

The positive rate of persistent CTCs (36.2%, 51 out of 141) is parallel to the rate of abnormal elevated serum CEA (33.3%, 47 out of 141) in our postoperative stages II–III colon cancer patients. Both of these two variables have significant roles in predicting early relapse of our studied patients either with univariate or multivariate analysis. Besides, postoperative high CEA level is also an independent predictor of the presence of persistent CTCs in our studied patients (Table 3). Previously, pre- and postoperative abnormal serum CEA levels in CRC patients have been documented as predictors of deeper local invasion of tumours, higher risk of occult metastases or higher rates of postoperative relapse (McCall *et al*, 1994; Wiratkapun *et al*, 2001). In fact, our recent work further indicated that molecular detection of postoperative CTCs is helpful in the early prediction of postoperative relapse in CRC patients with normal perioperative serum CEA levels, with a median leading time of 6 months before the measurement of elevated CEA values (Wang *et al*, 2007). Consequently, postoperative detection of CTCs would be a supplementary diagnostic tool to conventional serial check of serum CEA in the ‘early’ recognition of early relapse in stages II–III colon cancer patients receiving curative surgery.

Perineural invasion is a distinct pathologic entity, yet less common than lymphovascular invasion observed in CRC patients (Washington, 2008). As perineural invasion is often associated with high tumour grade and stage, it is thought of as an ominous prognostic sign in histological examination (Washington, 2008; Liebig *et al*, 2009b). Liebig *et al* (2009a, 2009b) reviewed and studied perineural invasion in CRC patients, and found that it was a strongly independent predictor of postoperative outcome in these patients. They indicated that among node-negative CRC patients, the 5-year disease-free survival rate for negative perineural invasion patients was almost three-fold greater than for positive perineural invasion cases (82 *vs* 29%, respectively) (Liebig *et al*, 2009a). Similar results were found for overall survival rate, where the 5-year overall survival rate for node-negative patients with positive perineural invasion was 43%, compared with 87% for patients without perineural invasion (Liebig *et al*, 2009a). Consistent with these investigations, this study also showed that perineural invasion is a significant independent predictor in detecting early relapse of stages II–III colon cancer patients.

According to the multivariate analysis, persistent postoperative CTCs is a more powerful predictor, either compared with conventional postoperative CEA level or with perineural invasion (Table 5). Actually, 11 patients in our study with normal postoperative CEA level and no histological perineural invasion are correctly predicted to have an early relapse by persistent CTCs. Therefore, detection of CTCs is superior to elevated CEA level or positive perineural invasion in identifying early relapse of postoperative stages II–III CRC patients. Although CTCs testing is currently more expensive than traditional CEA measurement or histological examination, it can be assumed that if multiple times point testing becomes more routine, a reduction in cost per test could be anticipated. Moreover, earlier detection of recurrent malignancy can bring more therapeutic benefits by detecting postoperative CTCs in patients and save more medical expenditures.

In summary, our study demonstrates that in addition to the check of perineural invasion and high postoperative CEA level, the persistent presence of postoperative CTCs via multiple blood sampling is a useful supplementary tool in detecting early relapse and survival rate of stages II–III colon cancer patients undergoing curative surgery. Certainly, there is a need for large, well-designed prospective trials to verify the clinical significance of CTCs in postoperative early relapse in CRC patients.

## Figures and Tables

**Table 1:** Clinicopathologic features of 141 stages II and III colon cancer patients undergoing curative resection

**Variables**	**Number (%)**
*Gender*	
Male/female	75(53.2)/66(46.8)
Age (y/o)	64.1±12.1
	
*Maximum size (cm)*	
<5/⩾5	68(48.2)/73(51.8)
	
*Location*	
Right-sided/left-sided^a^	58(41.1)/83(58.9)
	
*Depth of invasion*	
T_1_+T_2_/T_3_+T_4_	22(15.6)/119(84.4)
	
*Lymph node metastasis*	
Yes/no	91(64.5)/50(35.5)
	
*Vascular invasion*	
Yes/no	74(52.5)/67(47.5)
	
*Perineural invasion*	
Yes/no	78(55.3)/63(44.7)
	
*Histology*	
WD/MD/PD	10(7.1)/103(73.0)/28(19.9)
	
*Preoperative CEA (ng* *ml*^*−1*^*)*	
<5/⩾5	56(39.7)/85(60.3)
	
*Postoperative CEA (ng* *ml*^*−1*^*)*	
<5/⩾5	94(66.7)/47(33.3)
	
*Type of surgery*	
Laparotomy/laparoscopy-assisted	110(78.0)/31(22.0)
	
*Early postoperative relapse*	
Yes/no	48(34.0)/93(66.0)
	
*Type of tumor*	
Adenocarcinoma/mucinous	119(84.4)/22(15.6)
	
*Persistent postoperative CTCs*	
Yes/no	51(36.2)/90(63.8)
	
*Adjuvant therapy*	
Yes/no	110(78.0)/31(22.0)

Abbreviations: CEA=carcinoembryonic antigen; CTC=circulating tumor cell; MD=moderately differentiated; PD=poorly differentiated; WD=well differentiated.

a Right-sided, cecum+ascending colon+transverse colon; left-sided, descending colon+sigmoid colon.

**Table 2:** Correlation between persistent postoperative CTCs and clinicopathologic features of 141 stages II and III colon cancer patients using univariate analysis^a^

	**Persistent postoperative CTCs**		
	**Yes (*n*=51)**	**No (*n*=90)**	
	**No. (%)**	**No. (%)**	***P-*value^a^**
*Gender*			
Male/female	25(49.0)/26(51.0)	50(55.6)/40(44.4)	0.455
			
*Age (y/o)*			
<65/⩾65	31(60.8)/20(39.2)	56(62.2)/34(37.8)	0.866
			
*Maximum size (cm)*			
<5/⩾5	30(58.8)/21(41.2)	38(42.2)/52(57.8)	0.058
			
*Location*			
Right-sided/left-sided^b^	26(51.0)/25(49.0)	32(35.6)/58(64.4)	0.074
			
*Depth of invasion*			
T_1_+T_2_/T_3_+T_4_	4(7.8)/47(92.2)	20(22.2)/70(77.8)	0.029
			
*Lymph node metastasis*			
Yes/no	35(68.6)/16(31.4)	56(62.2)/34(37.8)	0.445
			
*Vascular invasion*			
Yes/no	38(74.5)/13(25.5)	36(40.0)/54(60.0)	<0.001
			
*Perineural invasion*			
Yes/no	37(72.5)/14(27.5)	41(45.6)/49(54.4)	0.002
			
*Histology*			
WD+MD/PD	44(86.3)/7(13.7)	68(75.6)/22(24.4)	0.130
			
*Preoperative CEA (ng* *ml*^*–1*^*)*			
<5/⩾5	14(27.5)/37(72.5)	39(46.7)/54(53.3)	0.025
			
*Postoperative CEA (ng* *ml*^*–1*^*)*			
<5/⩾5	24(47.1)/27(52.9)	70(77.8)/20(22.2)	<0.001
			
*Adjuvant therapy*			
Yes/no	43(84.3)/8(15.7)	67(74.4)/23(25.6)	0.174

Abbreviations: CEA=carcinoembryonic antigen; CTC=circulating tumor cell; MD=moderately differentiated; PD=poorly differentiated; WD=well differentiated;

a *χ*^2^ test.

b Right-sided, cecum+ascending colon+transverse colon; left-sided, descending colon+sigmoid colon.

**Table 3:** Correlation between persistent postoperative CTCs and clinicopathologic features of 141 stages II and III colon cancer patients using multivariate logistic regression analysis

**Variables**	**Coefficient**	**s.e.**	***P-*value**	**Hazard ratio^a^**
Depth (T3+T4/T1+T2)	0.699	0.627	0.264	3.012 (0.589–9.849)
Vascular invasion (yes/no)	1.006	0.430	0.019	2.732 (1.176–6.394)
Perineural invasion (yes/no)	0.496	0.433	0.252	1.642 (0.703–3.831)
Postoperative CEA (⩾5/<5)	0.999	0.404	0.013	2.717 (1.230–5.988)

Abbreviations:CTC=circulating tumor cells; CEA=carcinoembryonic antigen (ng ml^–1^); s.e.=standard error.

a Values in parentheses are 95% confidence intervals.

**Table 4:** Correlation between postoperative relapse pattern and clinicopathologic features of 141 stages II and III colon cancer patients using univariate analysis

	**Early relapse (*n*=48)**	**Non-early relapse/ No relapse (*n*=93)**	
	**No. (%)**	**No.** **(%)**	***P-*value** a
*Gender*			
Male/female	23(47.9)/25(52.1)	52(55.9)/41(44.1)	0.367
			
*Age (y/o)*			
<65/⩾65	28(58.3)/20(41.7)	59(63.4)/34(36.6)	0.554
			
*Maximum size (cm)*			
<5/⩾5	26(54.2)/22(45.8)	42(45.2)/51(54.8)	0.311
			
*Location*			
Right-sided/left-sided^b^	24(50.0)/24(50.0)	59(43.4)/34(36.6)	0.124
			
*Depth of invasion*			
T_1_+T_2_/T_3_+T_4_	5(10.4)/43(89.6)	19(20.4)/74(79.6)	0.134
			
*Lymph node metastasis*			
Yes/no	37(77.1)/11(22.9)	54(58.1)/39(41.9)	0.025
			
*Vascular invasion*			
Yes/no	34(70.8)/14(29.2)	40(43.0)/53(57.0)	0.002
			
*Perineural invasion*			
Yes/no	36(75.0)/12(25.0)	42(45.2)/51(54.8)	0.001
			
*Histology*			
WD+MD/PD	40(83.3)/8(16.7)	72(77.4)/21(22.6)	0.410
			
*Preoperative CEA (ng* *ml*^*−1*^*)*			
<5/⩾5	17(35.4)/31(45.6)	39(41.9)/54(58.1)	0.453
			
*Postoperative CEA (ng* *ml*^*−1*^*)*			
<5/⩾5	23(47.9)/25(52.1)	71(76.3)/22(23.7)	0.001
			
*Type of surgery*			
Laparotomy/ laparoscopy-assisted	32(66.7)/16(33.3)	78(83.9)/15(16.1)	0.019
			
*Type of tumor*			
Adenocarcinoma/ mucinous	43(89.6)/5(10.4)	76(81.7)/17(18.3)	0.223
			
*Adjuvant therapy*			
Yes/no	37(77.1)/11(22.9)	73(78.5)/20(21.5)	0.848
			
*Persistent postoperative CTCs*			
Yes/no	37(77.1)/11(22.9)	14(15.1)/79(84.9)	<0.001

Abbreviations: CEA=carcinoembryonic antigen; CTC=circulating tumor cell; MD=moderately differentiated; PD=poorly differentiated; WD=well differentiated.

a *χ*^2^ test.

b Right-sided, cecum+ascending colon+transverse colon; left-sided, descending colon+sigmoid colon.

**Table 5:** Correlation between postoperative early relapse and clinicopathologic features of 141 stages II and III colon cancer patients using multivariate cox proportional hazards regression analysis

**Variables**	**Coefficient**	**s.e.**	***P-*value^a^**	**Hazard ratio^b^**
Surgery (laparoscopy/laparotomy)	0.382	0.379	0.414	1.465 (0.697–3.077)
Lymph node metastasis (yes/no)	0.389	0.349	0.265	1.475 (0.745–2.923)
Vascular invasion (yes/no)	0.585	0.593	0.288	1.392 (0.402–3.786)
Perineural invasion (yes/no)	0.875	0.611	0.034	1.974 (1.290–3.861)
Postoperative CEA (⩾5/<5)	1.237	0.645	0.020	2.377 (1.273–4.255)
Persistent postoperative CTCs (yes/no)	1.832	0.613	<0.001	11.035 (4.396–32.190)

Abbreviations: CTC=circulating tumor cells; CEA=carcinoembryonic antigen (ng ml^–1^); s.e.=standard error.

a Cox proportional hazards regression analysis.

b Values in parentheses are 95% confidence intervals.
